# Enterococcal Membrane Vesicles as Vaccine Candidates

**DOI:** 10.3390/ijms242216051

**Published:** 2023-11-07

**Authors:** Theresa Maria Wagner, Felipe Romero-Saavedra, Diana Laverde, Mona Johannessen, Johannes Hübner, Kristin Hegstad

**Affiliations:** 1 Research Group for Host-Microbe Interactions, Department of Medical Biology, Faculty of Health Sciences, UiT The Arctic University of Norway, N-9037 Tromsø, Norway; theresa.wagner@uit.no (T.M.W.); mona.johannessen@uit.no (M.J.); 2 Division of Pediatric Infectious Diseases, Dr. von Hauner Children’s Hospital, Ludwig Maximilians University, 80337 Munich, Germany; frsquim@gmail.com (F.R.-S.); dianalaverde@gmail.com (D.L.); johannes.huebner@med.uni-muenchen.de (J.H.); 3 Norwegian National Advisory Unit on Detection of Antimicrobial Resistance, Department of Microbiology and Infection Control, University Hospital of North Norway, N-9038 Tromsø, Norway

**Keywords:** *Enterococcus faecium*, VRE, vaccine, bacterial membrane vesicles, infection control

## Abstract

*Enterococcus faecium* is a leading cause of nosocomial infections, particularly in immunocompromised patients. The rise of multidrug-resistant *E. faecium*, including Vancomycin-Resistant Enterococci (VRE), is a major concern. Vaccines are promising alternatives to antibiotics, but there is currently no vaccine available against enterococci. In a previous study, we identified six protein vaccine candidates associated with extracellular membrane vesicles (MVs) produced by nosocomial *E. faecium*. In this study, we immunized rabbits with two different VRE-derived MV preparations and characterized the resulting immune sera. Both anti-MV sera exhibited high immunoreactivity towards the homologous strain, three additional VRE strains, and eight different unrelated *E. faecium* strains representing different sequence types (STs). Additionally, we demonstrated that the two anti-MV sera were able to mediate opsonophagocytic killing of not only the homologous strain but also three unrelated heterologous VRE strains. Altogether, our results indicate that *E. faecium* MVs, regardless of the purification method for obtaining them, are promising vaccine candidates against multidrug-resistant *E. faecium* and suggest that these naturally occurring MVs can be used as a multi-antigen platform to elicit protective immune responses against enterococcal infections.

## 1. Introduction

*Enterococcus faecium* is a leading cause of nosocomial infections, especially in immunocompromised patients. Multidrug-resistance of *E. faecium*, including Vancomycin-Resistant Enterococci (VRE), impairs therapy and limits treatment options. The increasing incidence of VR*E. faecium* across Europe is of particular concern [1].

Treatment of enterococcal infections with currently available antimicrobials is often a challenge for clinicians due to its high intrinsic antimicrobial resistance, its capacity to acquire novel resistance genes, and its ability to withstand harsh conditions, including disinfectants [2,3,4].

Given the prevalence of enterococcal infections and the treatment challenges, there is an urgent need to develop new approaches to treat or prevent these infections. Immunotherapies, among other options, represent a promising avenue of research [5].

For enterococci, there are no available vaccines yet, even though research has been ongoing to identify vaccine candidates [6,7,8]. Several polysaccharide and protein antigens have been described as potential vaccine candidates against *Enterococcus faecalis* and *E. faecium* [5,6,7,8,9,10,11].

In a previous study, we characterized membrane vesicles (MVs) released by four nosocomial *E. faecium* strains and found that they are associated with six described protein vaccine candidates, among others [12]. Sera raised in rabbits against these protein antigens (SagA [6], PsaA, AdcA [7], PBP5, LysM, DdcP, and PpiC [8]) have been shown to mediate the opsonic killing against several enterococcal strains. Moreover, passive immunization with rabbit antibodies against these proteins significantly reduced the colony counts of *E. faecium* E155 in different mouse organs, indicating the effectiveness of these vaccine candidates in targeting different enterococcal pathogens [6,7,8].

MVs are promising vaccine candidates since they are highly stable, non-infectious, non-replicative particles. They contain major immunogenic proteins and are thus able to elicit responses in both arms of the immune system and display adjuvant activity [13,14]. The meningococcal serogroup B vaccine 4CMenB was the first MV-based vaccine to be licensed for human use [15]. This vaccine was first tested in Norway [16]. Also, in the Gram-positive pathogens *Streptococcus pneumoniae* [17] and *Staphylococcus aureus* [18,19], the protective effects of immunization with MVs were recently shown.

Taking together all our previous results, we aimed in the present study to immunologically characterize anti-MV sera to lead the way in the development of an MV-based VRE vaccine.

## 2. Results

### 2.1. Immunization with Enterococcal Membrane Vesicles Triggers an Immune Response

Isolation of MVs from 500 mL overnight (o.n) culture of *E. faecium* E155 in brain heart infusion (BHI) media with 8 mg/L vancomycin led to a yield of 376 ± 31 µg, and the MVs had a size of 107 ± 52 nm and 8.3 × 10^8^ ± 1.2 × 10^7^ particles/mL as measured in Nanosight and 148 ± 94 nm and 6.9 × 10^7^ ± 2.3 × 10^7^ particles/mL in ZetaView, which is comparable to our previous findings [12]. *E. faecium* E155 MVs did not show cytotoxicity at 1–100 ng per 2.5 × 10^4^ eukaryotic cells in cell lines representing pharynx epithelium, keratinocytes, and intestinal cells (Figure S1). For a monocyte cell line and human neutrophils, 1000 ng MV per well was tested in addition to 1–100 ng per 2.5 × 10^4^ cells. Only for neutrophils was cytotoxicity seen at higher MV-protein concentrations, which might be related to the fragility of these cells, whose cell death can be caused by agitation and centrifugation [20].

**Figure 1:**
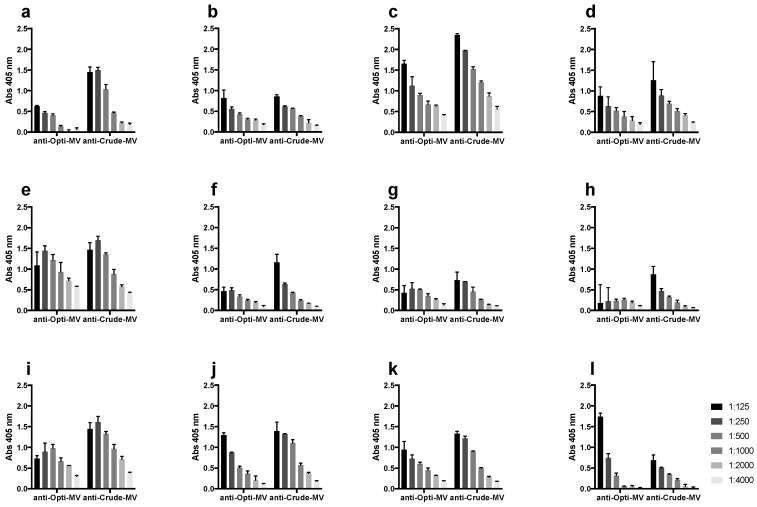
Immunoreactivity of anti-membrane vesicle (MV) sera against different *E. faecium* strains in ELISA at dilutions calculated as absorption (Abs). Abs 405 nm: _Terminal bleed_; Abs: 405 nm _Pre-bleed_; bars show the mean of triplicates with SD. (**a**) Homologous strain *E. faecium* E155, (**b**) VRE11236/1, (**c**) VRE757875, (**d**) VRE1.231.408, (**e**) K59-51, (**f**) K60-29, (**g**) K59-17, (**h**) K59-44, (**i**) KresEnt-1, (**j**) K59-26, (**k**) K59-20, and (**l**) 50939184. Opti-MV = OptiPrep-purified MVs.

The two different purified samples from *E. faecium* E155 triggered an immune response in rabbits and were as follows: (1) Crude, the MV pellet that was obtained after ultracentrifugation and (2) OptiPrep, the MV pellet obtained after ultracentrifugation and an additional density gradient centrifugation. IgM and IgG levels were quantified via an enzyme-linked immunosorbent assay (ELISA) (Table 1). In addition, IgG and IgM were titrated on adsorbed MVs, and the reactivity was 3.55 times higher in the sera raised against Crude compared to OptiPrep. Thus, the sera were normalized to have the same concentration as specific IgGs in the experimental assays. Both antisera showed high immunoreactivity in whole-bacterial-cell ELISA towards the homologous strain *E. faecium* E155 (Figure 1a), as well as three other VR*E. faecium* strains (Figure 1b–d). Whole-bacterial-cell ELISA with strains representing different STs of Clade A1 and B *E. lactis* showed binding in eight strains (K59-51 ST18, K60-29 ST19, K59-17 ST22, K59-44 ST32, KresEnt-1 ST80, K59-26 ST94, K59-20 ST203, 50939184 ST800) (Figure 1e–l). The seven vaccine candidates were present in the genomes of all strains used (Table S1 and Figure S2).

### 2.2. Anti-Enterococcal Membrane Vesicle Sera Mediate Opsonophagocytic Killing

**Figure 2:**
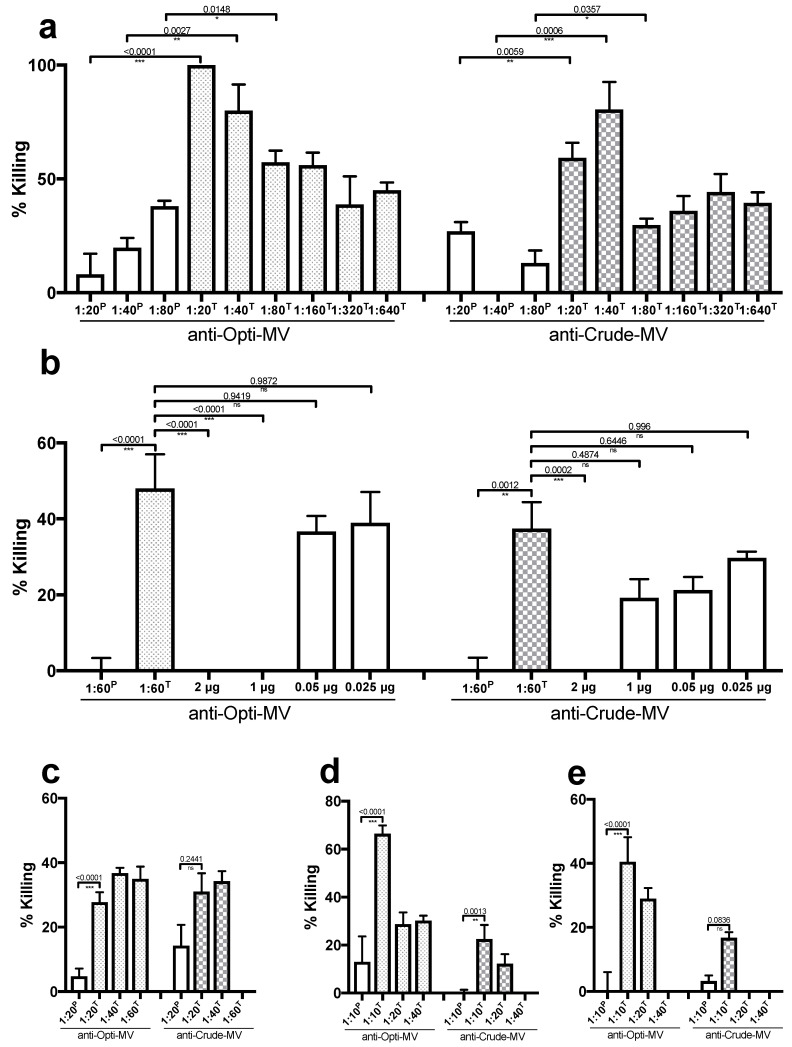
Opsonophagocytic killing activity of *E. faecium*. (**a**) Killing of the homologous strain E155 by anti-MV sera at dilutions 1:20 to 1:640 (statistical *p*-values were obtained with a two-tailed unpaired *t*-test, *n* = 4), *(***b**) opsonophagocytic inhibition assay with sera at a dilution of 1:60 and decreasing amounts of MVs (2–0.025 µg), (**c**) opsonophagocytic killing of the heterologous strain VRE11236/1, (**d**) opsonophagocytic killing of the heterologous strain VRE757875, (**e**) opsonophagocytic killing of the heterologous strain VRE1.231.408. Bars show mean (*n* = 4) according to scanning electron microscope (SEM). P indicates pre-bleed and T terminal bleed. Opti-MV = OptiPrep-purified MVs. Statistical *p*-values are given as calculated with a two-tailed unpaired *t*-test, *n* = 4, (<0.0001 ***, 0.0002 **, 0.05 *, >0.05 ns).

The terminal bleed of both anti-MV sera showed significantly higher opsonophagocytic killing activity towards the homologous *E. faecium* strain E155 compared to the pre-bleed (Figure 2a), while the controls with only complement, or only polymorphonuclear neutrophils (PMNs) did not show a killing rate above 10%. The anti-Crude and anti-OptiPrep mediated killing at a dilution of 1:60, where the rate of killing was 40–50%, and could be blocked by the addition 2 µg of the antigen (Crude MVs or OptiPrep MVs), confirming the specificity of the killing (Figure 2b). The blocked killing could be restored by dilution of the antigen to 0.025 µg. This was also reproducible when anti-Opti-MV-sera were incubated with Crude MVs and anti-Crude-MV sera were incubated with OptiPrep-purified MVs, showing that all the important epitopes were conserved in the different preparations of the MVs (Figure S3). The opsonophagocytic killing (OPA)/opsonophagocytic inhibition (OPIA) assays were confirmed with two individual freezer stocks of the homologous *E. faecium* strain E155 and with PMNs isolated from different donors. In addition to the homologous strain, opsonophagocytic killing activity was shown in unrelated heterologous VR*E. faecium* strains (Figure 2c–e). It was not possible to evaluate the opsonophagocytic killing activity of the anti-MV with several other *E. faecium* strains because the results were inconclusive (OPA controls failed) as these strains were sensitive to either pre-existing antibodies, complement, and/or PMNs, or displayed agglutination in the presence of serum (Table S2), as previously described for other *E. faecium* strains [21].

## 3. Discussion

The clinical management of enterococcal infections is increasingly challenging due to the emergence of multidrug-resistant isolates. The increasing abundance of VR*E faecium* across Europe is particularly worrying [1]. Resistance to last-resort antibiotics is emerging [22], which drastically limits treatment options. Multidrug-resistant (MDR) *E. faecium* infections are associated with a high economic burden and higher morbidity and mortality rates compared to infections caused by susceptible strains [23,24]. Moreover, enterococci are highly persistent due to their ability to withstand harsh conditions, resist biocides, their biofilm-forming ability, and their high genetic malleability [2,25,26], which challenges decontamination efforts. Thus, enterococci are an important source of nosocomial outbreaks and pools of resistance gene spread. To address the gap in treatment options, vaccines represent promising strategies to expand the current panel of available treatments and prevention measures. Vaccine development approaches offer potential benefits in terms of efficacy, safety, and long-term cost-effectiveness for the management of enterococcal infections [27].

Amongst innovative vaccine strategies, the use of bacterial (outer) membrane vesicles ((O)MVs) stands out since they combine the advantages of natural mimicry, broad antigenic coverage, enhanced immunogenicity, safety, and ease of production. Since (O)MVs are not capable of self-replication but still mimic the immunogenic properties of the (O)MV-producing bacterium, they are an attractive multi-antigen vaccine platform [28,29]. One OMV-based vaccine, 4cMenB protective against *Neiserria meningitidis* [15], has been in use for several years. (O)MVs of other bacteria have been investigated for their potential as vaccines against infection, such as against *Bordetella pertussis* [30], *Vibrio cholerae* [31], *Escherichia coli* [32], *Haemophilus parasuis* [33], *Pseudomonas aeruginosa* [34], *Acinetobacter baumannii* [35], *Salmonella* [36], *S. pneumoniae* [37], *S. aureus* [38], and others [29]. This is the first study to show the potential of enterococcal MVs as vaccine candidates in the fight against VRE.

The isolation and content of enterococcal MVs have been described in both *E. faecium* [12] and *E. faecalis* [39]. In a previous study [12], we found that *E. faecium* MVs are associated with the characterized vaccine candidates SagA [6], PsaA, AdcA [7], PBP5, LysM, DdcP, and PpiC [8]. Antisera raised against the recombinant SagA, which has been described as a major secreted *E. faecium* antigen able to bind extracellular matrix proteins such as fibrinogen, collagen type I and IV, fibronectin, and laminin [40], showed specific opsonic killing by white blood cells in vitro [6]. In a mouse bacteremia model, a significant reduction in VRE (*E. faecium* E155) colony-forming unit (CFU) count in blood was shown upon passive immunization with anti-SagA serum [6]. Rabbit polyclonal antibodies raised against the four purified surface-exposed proteins, PBP5 (a low-affinity penicillin-binding protein 5), LysM (a basic membrane lipoprotein, a peptidoglycan-binding protein), DdcP (a D-alanyl-D-alanine carboxypeptidase), and PpiC (a peptidyl-prolyl cis-trans isomerase) mediated specific opsonic killing of the homologous strain *E. faecium* E155 as well as four other clinical strains [8]. The CFU count of E155 was significantly reduced in a bacteremia model when mice were passively immunized with the individual antisera. Also, rabbit polyclonal antiserum against the purified metal-binding lipoprotein PsaA (a manganese ABC transporter substrate-binding lipoprotein) mediated specific opsonic killing of the homologous strain *E. faecium* E155 as well as four other clinical strains, and passive immunization reduced CFU count in a mouse bacteremia model [7]. To broaden vaccine coverage and enhance efficiency, conjugates of polysaccharide and proteinaceous virulence factors, where the protein acts as a carrier and immunogen simultaneously, have been proposed [41]. Two protein vaccine candidates, SagA and PpiC, were used as antigens and carrier proteins for the enterococcal polysaccharide diheteroglycan. These two glycoconjugates showed cross-reactivity in an ELISA and opsonophagocytic assay against several clinical *E. faecium* and *E. faecalis* strains, as well as protective effects in a passive immunization mouse sepsis model [21]. Yet, the combination of several protein vaccine candidates in a multi-antigen preparation has not been evaluated [27]. In this study, the MVs act as vehicles for presenting multiple antigens. Unlike multi-protein vaccines, the purification process for MVs only requires one step to isolate several antigens simultaneously. Additionally, the antigens are naturally present in the MV at a similar ratio. As a result, it is reasonable to assume that the likelihood of immune escape against this proposed polyvalent vaccine is lower than that of monovalent vaccines.

We used two differently purified MV preparations, one Crude and one gradient-purified (OptiPrep) preparation. Since the IgG concentration was 3.55 times higher in the sera raised against Crude, the sera were normalized for the specific IgG concentration in the experimental assays. We speculate the slightly “cleaner” OptiPrep sample elicited a slightly less intense immune response. We also found that both preparations resulted in the desired IgG production and the Crude MV preparation did not show cytotoxicity.

In the immunosorbent assay, it was shown that both sera, anti-OptiPrep and anti-Crude, bind the homologous strain, three other VRE strains, and strains of other STs. Of these strains, K59-51 ST18, K59-20 ST203, and KresEnt-1 ST80 represent STs which are clinically relevant in Europe [42]. The MV-induced sera thus have the potential to protect against a broad range of clinical *E. faecium* strains, including VRE. The observed broad cross-reactivity of anti-MV sera to several VRE strains is in line with what has previously been observed for the other vaccine candidates [8]. Moreover, the anti-MV sera showed similar opsonic killing properties when compared to the single-protein enterococcal vaccine candidates contained in the MVs, such as SagA [6], PsaA, AdcA [7], PBP5, LysM, DdcP, and PpiC [8].

In summary, this study describes *E. faecium* MVs as promising multi-antigen, easy-to-produce vaccine candidates, which can be utilized as an alternative strategy in the infection control of MDR *E. faecium*.

## 4. Materials and Methods

### 4.1. Bacterial Strains

The bacterial strains used in this study were *E. faecium* E155 [43], from which MVs were isolated, as in [12]; 3 VRE strains, VRE757857 [44], VRE11236/1 [21], and VRE1.231.408 [45]; and *E. faecium* strains representing different sequence types (STs), for which detailed information is given in Supplementary Table S3.

### 4.2. Isolation and Characterization of Membrane Vesicles

MVs were purified from the supernatant of *E. faecium* E155 through ultracentrifugation, as described in [12]. In a pilot study, it was established which yield could be obtained under different growth and purification conditions (Supplementary Methods and Figure S4).

In the final protocol, 20 mL overnight (o.n) culture of *E. faecium* E155 was used to inoculate 1 L of BHI with 8 mg/L vancomycin in a 2 L winged flask and incubated at 37 °C with 220 rpm shaking for 16 h to an OD_600nm_ 2.7 ± 0.1. Bacterial cells were removed via centrifugation at 6000× *g* for 30 min (JLA 9.1000 rotor, Beckman Instruments Inc., Fullerton, CA, USA) and the supernatant was filtrated through a 0.45 µm followed by a 0.22 µm pore filter (Stericup-GP, PVDF membrane, Merck Millipore, Burlington, MA, USA). The sterile supernatant was ultracentrifuged at 30,000 rpm at 4 °C for 4 h (45 TI rotor), and the obtained pellet was washed with phosphate-buffered saline (PBS) and ultracentrifuged at 30,000 rpm at 4 °C for 3 h (SW 50.1 rotor). The washed MV pellet at this stage was referred to as “Crude” and stored at −80 °C or purified further.

Some of the Crude MV pellets were subjected to density gradient centrifugation. They were mixed with an equal volume 60% OptiPrep solution (iodixanol in water, D1556 Sigma-Aldrich, Saint-Louis, MO, USA) to obtain a 30% solution. A total of 1 ml of the 30% sample OptiPrep solution was pipetted in the bottom of an ultracentrifuge tube (Thinwall, Ultra-ClearTM, 5 mL, 13 × 51 mm Beckman Coulter Centrifuge Tube) and overlayed with 2 mL of 25% and 1 mL of 5% OptiPrep solution in PBS and ultracentrifuged at 30,000 rpm at 4 °C for 3 h (SW 50.1 rotor) with slow acceleration and deceleration. The MV-containing fraction, whose ring formation was visible, was washed in a filter to remove the OptiPrep solution (10 kDa molecular weight cut-off, Vivaspin (Sartorius Göttingen, Germany) at 4000 *g* for 10 min at 4 °C, resuspended in PBS, and stored at −80 °C. The MV pellet obtained after density gradient centrifugation was referred to as “OptiPrep”.

The protein content of the samples was quantified using a Qubit fluorometer (Thermo Fisher Scientific, Waltham, MA, USA) and analyzed in Nanosight (NTA Version 3.0 0060, SOP standard measurement, SCMOS, Malvern Instruments, Malvern, UK) and ZetaView (ZetaVIEW S/N 21-668, Software ZetaView (version 8.05.14 SP7), 488 nm, Scatter, Particle Metrix GmbH, Inning am Ammersee, Germany).

The cytotoxicity of the MVs towards three different cell types was evaluated by measuring the release of lactate dehydrogenase (LDH). Two pharynx epithelial cell lines (Fadu HTB43 (American type culture collection (ATCC, Rockville, MD, USA)) and Detroit 562 (ATCC) in Dulbecco’s Modified Eagle’s Medium (DMEM)–high glucose (Sigma-Aldrich) 10% fetal bovine serum (FBS) (Sigma-Aldrich)), a keratinocyte cell line from human skin (HaCat (ATCC) in DMEM-low glucose (Sigma-Aldrich) 10% FBS), a large intestine cell line (CaCo (ATCC) in Minimum Essential Medium Eagle high glucose (Sigma-Aldrich) 10% FBS and 1% non-essential amino acids), a monocyte cell line (Thp1 (ATCC) in Thp1 medium with 10% FBS and 25 nM phorbol-12-myristate-13-acetate (Sigma-Aldrich)), and neutrophils (isolated from the fresh human blood of a healthy volunteer, isolated via Polymorphprep (Axis-Shield density gradient, Gentaur Europe, Kampenhout, Belgium), in Roswell Park Memorial Institute (RPMI)-1640 (Sigma-Aldrich) with 0.05% human serum albumin) were seeded at 2.5 × 10^4^ cells/well 24 h prior to the experiment (36 h for Thp1 cells for differentiation and directly for neutrophils) in 96-well plates and incubated at 37 °C with 5% CO_2_. Crude MVs (1 to 1000 ng per well) up to 10 µL in PBS were added, and samples were taken at time points 1, 3, and 6 h. LDH was measured using the Cytotoxicity Detection Kit (Roche, Basel, Switzerland), where the red product formazan was measured at 490 nm. Cytotoxicity was calculated as cytotoxicity (%) = ((experimental value − low control) − (high control − low control)) × 100, where the low control is untreated cells and the high control is cells treated with lysis buffer to achieve maximal LDH release.

### 4.3. Rabbit Immunizations

New Zealand White Rabbits were immunized with MVs with the following schedule: injections were conducted intramuscularly with 10 µg of MV + Freud’s incomplete adjuvant on days 14, 35, and 49. Bleeds were taken at days 0 (pre-bleed 1), 7 (pre-bleed 2), 42 (test-bleed), and 56 (terminal bleed).

Pre-bleeds were tested for their immunoreactivity towards the homologous strain *E. faecium* E155, and animals with the lowest reactivity were selected.

### 4.4. Specific Titer Quantification

ELISAs were performed as described in [21]. Microtiter plates (MaxiSorp 96, Sigma-Aldrich) were coated with the different antigen MVs (Crude or OptiPrep) as follows. A solution of 1 µg of the corresponding antigen in 100 µL of 0.2 M sodium carbonate/bicarbonate buffer, pH 9.4, was added to each well and incubated o.n at 4 °C. Washing steps were performed with 1 × PBS containing 0.05% Tween 20. Plates were blocked with 200 μL of 1 × PBS containing 3% bovine serum albumin (BSA) for 1 h at room temperature. Each antiserum in 100 µL in dilutions from 1:125 to 1:4000 was added in triplicate to antigen-coated wells and incubated for a further 1 h at room temperature. A goat anti-rabbit IgG alkaline phosphatase conjugate (Sigma-Aldrich) diluted to 1:1000 in 1 × PBS + 1% BSA was used as the secondary antibody, and p-nitrophenyl phosphate at 1 mg/mL in glycine buffer was used as the substrate (Sigma-Aldrich). After 1 h incubation at room temperature, the absorbance was measured at 405 nm in a BioTek Synergy H1 hybrid reader (Agilent Technologies, Santa Clara, CA, USA). Titers were calculated as follows: for each serum sample, the linear relationship between the OD and the log10[dilution factor] was used to extrapolate the intercept of an absorbance of 0.3 for each test, and this was taken as the ELISA endpoint titer [21].

### 4.5. IgG and IgM Titer Quantification

Total rabbit IgG and IgM were quantified as previously described [21]. Nunc-immuno Maxisorp 96 MicroWell plates (Thermo Fisher Scientific) were coated by adding 100 µL of either unlabeled anti-rabbit IgG or anti-rabbit IgM (Sigma-Aldrich) at a concentration of 1 µg/mL in a coating buffer (15 mM sodium carbonate, 35 mM sodium bicarbonate, pH 9.6). The plates were incubated on at 4 °C. Thereafter, wells were washed three times with 200 µL of a washing buffer (WB) containing 0.9% sodium chloride and 0.1% Tween 20 (Sigma-Aldrich). Subsequently, the wells were blocked by adding 200 µL of a blocking buffer (BB) containing 3% BSA (Carl Roth, Karlsruhe, Germany) in PBS and incubated for 2 h at room temperature. After the incubation, the wells were washed three times with 200 µL of WB. Next, 100 µL of the sample or standard dilutions were added in triplicates to the wells. For the standards, dilutions of either normal rabbit IgG or normal rabbit IgM ranging from 31.2 ng/mL to 0.12 ng/mL were prepared in BB. The sera to be tested (samples) were diluted in BB at dilutions ranging from 1:1,000,000 to 1:50,000,000. After adding the standards and samples, the plates were incubated for 2 h at room temperature, followed by three washes with 200 µL of WB. Then, 100 µL of either anti-rabbit IgG or anti-rabbit IgM alkaline phosphatase-conjugated produced in goat (Sigma-Aldrich), diluted to 1:1000, was added as the secondary antibody. The plates were incubated for an additional 2 h at room temperature, and the wells were washed four times with 200 µL of WB. Finally, the detection was performed as described above in the specific titter quantification section using p-nitrophenyl phosphate (Sigma-Aldrich) as a substrate. IgG and IgM concentrations in the samples were calculated against calibration curves generated with standard rabbit IgG or IgM dilutions.

### 4.6. Whole-Bacterial-Cell ELISA

Whole-bacterial-cell ELISA assays were performed as described [21]. In brief, bacteria were grown to OD_650nm_ 0.4 in 50 mL of tryptic soy broth (TSB) (Carl Roth) and pelleted. The bacterial pellet was washed twice in PBS, incubated in 25 mL 8% paraformaldehyde (Sigma-Aldrich) for 1 h at 4 °C, washed twice in PBS, and resuspended in a coating buffer (0.2 M sodium carbonate/bicarbonate buffer, pH 9.4) (Carl Roth). Microtiter plates (MaxiSorp 96) were coated with 100 µL of the bacterial suspension and incubated o.n at 4 °C. The ELISA was performed as described above under immunoglobulin titer quantification. The immunoreactivity was calculated as the ratio of the absorbance of the terminal immune serum to the absorbance of the pre-immune serum.

### 4.7. OPA/OPIA

The ability of anti-MV sera to mediate the opsonophagocytic killing of different *E. faecium* strains was evaluated with an OPA, and the specificity of this killing was evaluated with an OPIA, as described in [46].

For OPA, PMNs were freshly isolated with heparin–dextran (Carl Roth) from healthy adult volunteers and resuspended in RPMI (Thermo Fisher Scientific) with 15% FBS at 2 × 10^7^ cells/mL. Baby rabbit serum (Cedarlane, Hornby, ON, Canada) at a 1:30 dilution was used as a source of complement, and rabbit serum against AdcA [7] at a 1:25 dilution in RPMI with 15% FBS as a positive control. Bacteria were grown in TSB to mid-exponential phase (OD_650_ = 0.4), and 1 mL was pelleted and resuspended in RPMI with 15% FBS and diluted in RPMI with 15% FBS to achieve a concentration of 8 × 10^5^ CFU/mL. Equal volumes of bacterial suspension (8 × 10^5^ CFU/mL), PMNs (2 × 10^7^ cells/mL), complement source (7.5% final concentration), and anti-serum (anti-Crude, anti-OptiPrep, or anti-AdcA in RPMI with 15% FBS) dilutions were combined and incubated on a rotor rack at 37 °C for 90 min. After incubation, live bacteria were quantified using agar culture of serial dilutions. Percent killing was calculated by comparing the CFU counts of a sample not containing PMNs to the CFU counts of a sample with PMNs. For each assay, the following four controls without sera were included: (1) bacteria only, (2) bacteria and complement only, (3) bacteria and PMNs only, (4) bacteria and complement and PMNs only.

For OPIA, the antisera were incubated at 4 °C for 16 h on a rotor rack with the corresponding inhibitor MV (Crude or OptiPrep) at concentrations ranging between 0.025 and 2 µg. Anti-serum incubated with PBS was used as a negative control. Subsequently, the antisera were used as described for OPA.

### 4.8. Data Analysis

Data were analyzed and visualized in GraphPad Prism 7.0e. The absorbance (Abs) detected via a whole-cell ELISA was expressed as the mean (*n* = 3) with an SD of 405 nm _Terminal bleed_ − Abs 405 nm _Pre-bleed_. The percentage of opsonophagocytic killing was expressed as the mean (*n* = 4) with SEM, and statistical significance was calculated using a two-tailed unpaired *t*-test. *p*-values of <0.05 were considered statistically significant (<0.0001 ***, 0.0002 **, 0.05 *, >0.05 not significant (ns).

## Figures and Tables

**Table 1:** Total IgM and IgG levels in the two antisera during rabbit immunizations. Values are given in mg/mL.

		IgM (mg/mL)		IgG (mg/mL)	
Day	Bleed	Anti-OptiPrep	Anti-Crude	Anti-OptiPrep	Anti-Crude
0	1st Pre	0.70	0.40	8.39	7.15
7	2nd Pre	0.46	0.57	3.85	5.32
42	Test	0.90	0.37	7.42	5.53
56	Terminal	0.65	0.32	9.4	10.91

## Data Availability

Data are contained within the article and Supplementary Materials.

## Supplementary Materials

The following supporting information can be downloaded at: https://www.mdpi.com/article/10.3390/ijms242216051/s1.
