# Supplementary material for: Enterococcal Membrane Vesicles as Vaccine Candidates
Source: Int J Mol Sci. 2023 Nov 7;24(22):16051. doi: 10.3390/ijms242216051 (PMC10671723; doi:10.3390/ijms242216051)
Supplement: Supplementary file 1 [file ijms-24-16051-s001.zip › ijms-2684913-supplementary.pdf]

Supplementary

Enterococcal Membrane Vesicles as Vaccine Candidates

Theresa Maria Wagner, Felipe Romero-Saavedra, Diana Laverde, Mona Johannessen, Johannes Hübner, Kristin Hegstad

Supplementary Tables

Table S1. Presence of vaccine candidates in whole genome sequences of the strains used in this study and binding in whole cell enzyme-linked immunosorbent assay (ELISA) to anti-OptiPrep and anti-Crude as measured in ELISA.

**Gene**<br><br>**Locus**	***psaA***<br><br>E155_ 02566	***adcA***<br><br>E155_ 00526	***pBP5***<br><br>E155_ 02659	***lysM***<br><br>E155_ 01532	***ddcP***<br><br>E155_ 02369	***ppiC***<br><br>E155_ 01118	***sagA***<br><br>E155_ 00236	**ΔOpti 1:125**	**ΔCrude 1:125**
E155	x	x	x	x	x	x	x	0.6	1.5
K60-29	x[#]	x*	x	x	x	x	x	0.5	1.2
K59-17	x[#]	x*	x	x	x	x	x	0.4	0.7
K59-44	x[#]	x*	x	x	x	x	x	0.2	0.9
KresEnt-1	x[#]	x*	x	x	x	x	x	0.7	1.4
K59-26	x[#]	x*	x	x	x	x	x	1.3	1.4
K59-20	x	x	x	x	x	x	x	1.0	1.3
50939184	x[#]	x*	x	x	x	x	x	1.7	0.7

x indicates the presence, / indicates the absence of a gene, different variants are indicated as: [#] shorter version of *psaA* (nt1-123 and nt917-951 are missing), * shorter version of *adcA* (nt1-118 is missing); ΔOpti and ΔCrude give the difference between the terminal bleed and the pre-bleed at a dilution of 1:125 as measured in ELISA.

Table S2. Behavior of additional *E. faecium* strains in opsonophagocytic killing assay (OPA).

**Name**	**Collection**
K59-51	Sensitive to neutrophils, complement and pre-bleeds
K60-29	Sensitive to complement
K59-17	Sensitive to complement
KresEnt-1	Agglutination
K59-26	Sensitive to complement
K59-20	Sensitive to complement, agglutination
50939184	Sensitive to complement

Table S3. Characteristics of strains used in this study.

**Name**	**Collection/Ref**	**Source**	**ST**	**Clade**	**Year**	**Country**	**Isolation site**
E155	[43]	Hospitalized patient	17	A1	1995	US	Faeces
K59-51	VRE Study	Hospitalized patient	18	A1	2008	NOR	Blood
K60-29	VRE Study	Hospitalized patient	19	A1	2008	NOR	Blood
K59-17	VRE Study	Hospitalized patient	22	A1	2008	NOR	Blood
K59-44	VRE Study	Hospitalized patient	32	A1	2008	NOR	Blood
KresEnt-1	K-res	Hospitalized patient	80	A1	2014	NOR	Urine
K59-26	VRE study	Hospitalized patient	94	B, *E. latics*	2008	NOR	Blood
K59-20	VRE Study	Hospitalized patient	203	A1	2008	NOR	Blood
50939184	Tromsø 7	Non-hospitalized person	800	B, *E. latics*	2015	NOR	Faeces
VRE757857	[44]	Hospitalized patient	n.a[a]	n.a	n.a	US	n.a
VRE11236/1	[21]	Hospitalized patient	n.a	n.a	n.a	DE	Faeces
VRE1.231.408	[45]	Hospitalized patient	582	A1	2005	US	Blood

[a] US: the United States, NOR: Norway, DE: Germany, n.a: Information not available.

Supplementary Figures

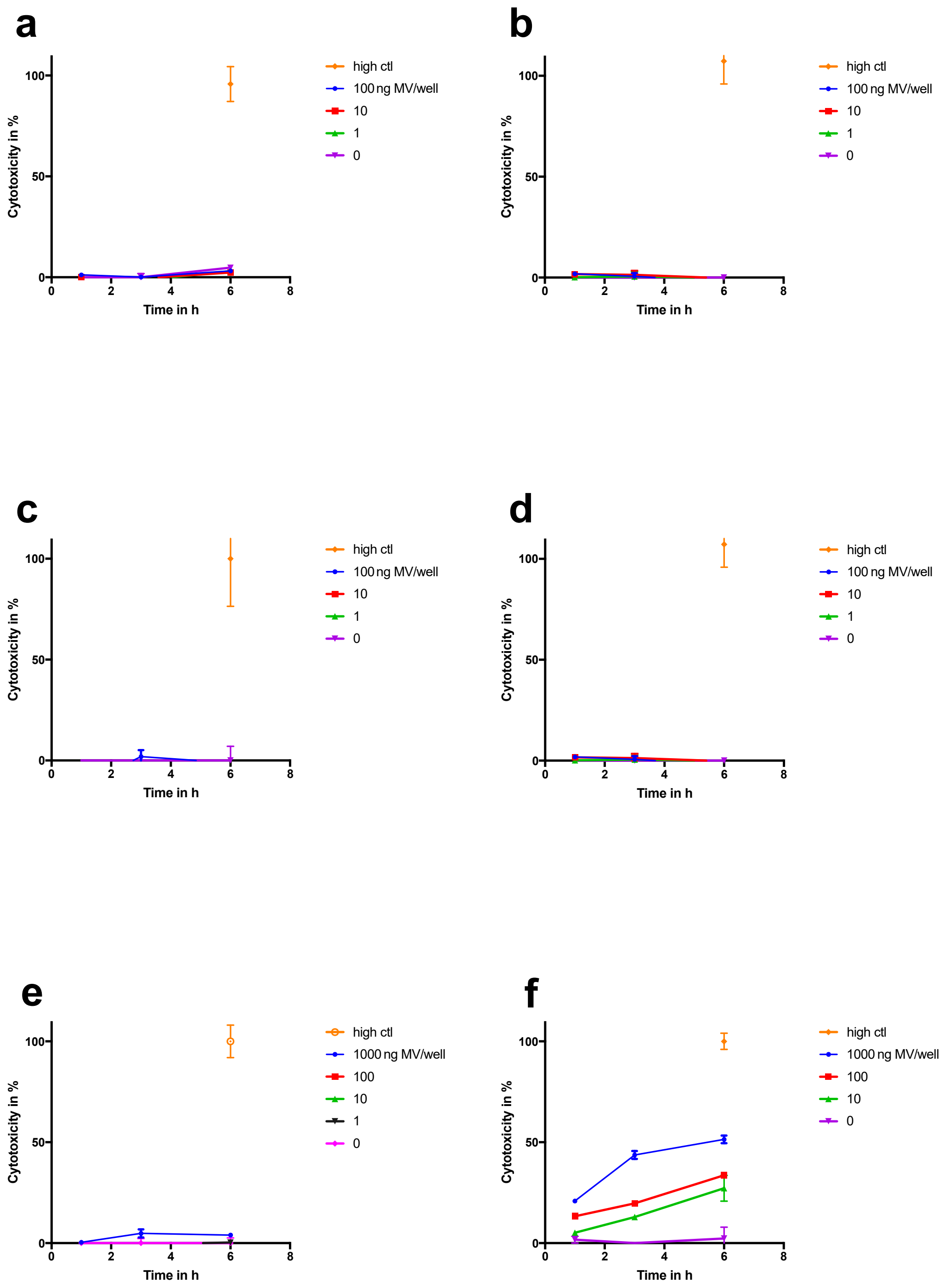

Figure S1. Cytotoxicity of Crude *E. faecium* E155 membrane vesicles (MVs) to different eukaryotic cells at 100 ng, 10 ng and 1 ng per well with 2.5×10^4 cells measured by lactate dehydrogenase (LDH) release. **(a)** FaDu pharynx epithelial cell line, **(b)** Detroit pharynx epithelial cell line, **(c)** HaCat keratinocyte cell line, **(d)** CaCo large intestine cell line, **(e)** Thp1 monocyte cell line, **(f)** Neutrophils isolated from fresh human blood. High ctl = cell lysed with lysis buffer for maximal LDH release. For Thp1 and neutrophils 1000 ng MV/ well were included. Symbols show mean (n_{well}=6) with scanning electron microscope (SEM*)*.

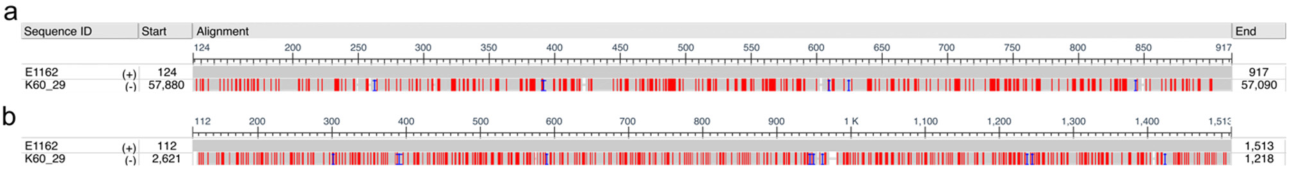

Figure S2. Alignment of *psaA* and *adcA* variants. a) *psaA* in E1162 and in K60-29 K59-27. B) *adcA* in E1162 and in K60-29. Mismatches are shown in red, and insertions relative to the anchor sequence are indicated by a blue bracket, visualized in National Center for Biotechnology Information Multiple Sequence Alignment Viewer 1.23.0.

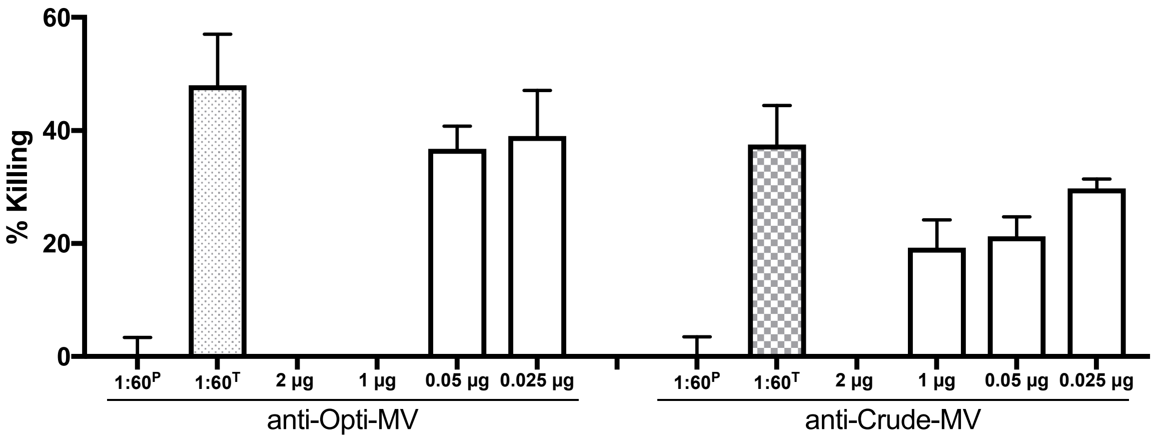

Figure S3. Opsonophagocytic inhibition assay with sera at a dilution of 1:60 and decreasing amounts of MVs (2-0.025 µg) where anti-Opti-MV-sera was incubated with Crude MV and anti-Crude-MV-sera was incubated with Opti MV. Bars show mean (n=4) with SEM. P indicates pre-bleed and T terminal bleed. Opti = OptiPrep purified MVs, Crude = Crude MVs.

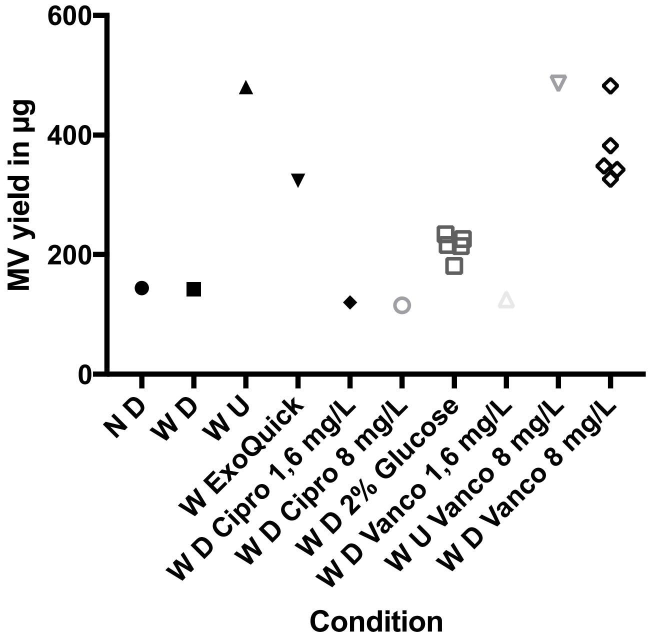

Figure S4. Crude MV yield in different conditions from 500 ml culture. N: normal flask, W: wide flask, U: up-concentration step, D: direct, Cipro: ciprofloxacin, Vanco: vancomycin. MV yield is measured after the final washing step in phosphate buffered saline (PBS), measured in Qubit.

Supplementary Methods

Optimization of MV production.

In addition to the described final protocol, the following conditions were tested. A normal 2 L growth flask was compared to a 2 L wide-winged flask; the direct method (as specified in the final protocol) was compared to the up-concentration method (sterile filtrated supernatant is concentrated using Amicon tubes (Merck Millipore, cut-off 100 kDa) in a Beckman centrifuge at 4000 ×*g* for 30 min at 4 °C) or ExoQuick (EQPL10TC-1, Sanbio); and Brain heart infusion (BHI) was compared to BHI supplemented with ciprofloxacin 1.6 or 8 mg/L or vancomycin 1.6 or 8 mg/L or 2% glucose. In all conditions, 20 ml overnight culture of *E. faecium* E155 was used to inoculate 1 L of BHI (or BHI supplemented with ciprofloxacin 1.6 or 8 mg/L or vancomycin 1.6 or 8 mg/L or 2% glucose) in a 2 L winged flask or a normal growth flask and incubated at 37 °C with 220 rpm shaking for 16 h. Bacterial cells were removed by centrifugation at 6000 *g* for 30 min (JLA 9.1000 rotor, Beckman Instruments Inc) and the supernatant was filtrated through a 0.45 μm followed by a 0.22 μm pore size filter (Stericup-GP, PVDF membrane, Millipore). The sterile supernatant was concentrated using Amicon tubes or ExoQuick or directly ultracentrifuged at 30.000 rpm at 4 °C for 4 h (45 TI rotor), and the obtained pellet was washed with PBS and ultracentrifuged at 30.000 rpm at 4°C for 3 h (SW 50.1 rotor).
